# The annual cycle for whimbrel populations using the Western Atlantic Flyway

**DOI:** 10.1371/journal.pone.0260339

**Published:** 2021-12-31

**Authors:** Bryan D. Watts, Fletcher M. Smith, Chance Hines, Laura Duval, Diana J. Hamilton, Tim Keyes, Julie Paquet, Lisa Pirie-Dominix, Jennie Rausch, Barry Truitt, Brad Winn, Paul Woodard

**Affiliations:** 1 Center for Conservation Biology, William & Mary, Williamsburg, Virginia, United States of America; 2 Non-Game Conservation Section, Wildlife Resources Division, Georgia Department of Natural Resources, Brunswick, Georgia, United States of America; 3 Mount Allison University, Sackville, New Brunswick, Canada; 4 Canadian Wildlife Service, Environment and Climate Change Canada, Sackville, New Brunswick, Canada; 5 Canadian Wildlife Service, Environment and Climate Change Canada, Iqaluit, Nunavut, Canada; 6 Canadian Wildlife Service, Environment and Climate Change Canada, Yellowknife, Northwest Territories, Canada; 7 The Nature Conservancy’s Volgenau Virginia Coast Reserve, Nassawadox, Virginia, United States of America; 8 Manoment Inc., Manomet, Massachusetts, United States of America; MARE – Marine and Environmental Sciences Centre, PORTUGAL

## Abstract

Many long-distance migratory birds use habitats that are scattered across continents and confront hazards throughout the annual cycle that may be population-limiting. Identifying where and when populations spend their time is fundamental to effective management. We tracked 34 adult whimbrels (*Numenius phaeopus*) from two breeding populations (Mackenzie Delta and Hudson Bay) with satellite transmitters to document the structure of their annual cycles. The two populations differed in their use of migratory pathways and their seasonal schedules. Mackenzie Delta whimbrels made long (22,800 km) loop migrations with different autumn and spring routes. Hudson Bay whimbrels made shorter (17,500 km) and more direct migrations along the same route during autumn and spring. The two populations overlap on the winter grounds and within one spring staging area. Mackenzie Delta whimbrels left the breeding ground, arrived on winter grounds, left winter grounds and arrived on spring staging areas earlier compared to whimbrels from Hudson Bay. For both populations, migration speed was significantly higher during spring compared to autumn migration. Faster migration was achieved by having fewer and shorter stopovers *en route*. We identified five migratory staging areas including four that were used during autumn and two that were used during spring. Whimbrels tracked for multiple years had high (98%) fidelity to staging areas. We documented dozens of locations where birds stopped for short periods along nearly all migration routes. The consistent use of very few staging areas suggests that these areas are integral to the annual cycle of both populations and have high conservation value.

## Introduction

Each year billions of birds make seasonal movements in order to improve survival and breeding success [1]. Breeding areas, winter areas, migratory staging areas and the routes that link them together represent an interconnected network and a set of constraints that imposes structure on the annual cycle of migrants. The set of constraints itself is in dynamic flux exerting pressure on migrants to adjust and reshape migratory strategies over time [e.g., 2–4]. Because connections within migratory networks are directional, conditions within one area may carry over and influence the energy budget or dictate the movement phenology within downstream areas [e.g., 5, 6]. Identifying these interdependencies requires an understanding of the basic architecture of the network. Mapping the annual cycle is a first step toward identifying critical nodes and bottlenecks throughout the network and understanding how migration systems have evolved and currently operate [7].

Hundreds of shorebird populations are experiencing ongoing declines on a global scale [8–10]. Population declines are particularly dramatic for species using the Western Atlantic Flyway with 65% of populations with recent trend data believed to be declining [11]. Among others, suggested causes for declines include mortality related to hunting [12, 13] or increased predation pressure [14, 15] impacts on foraging within staging areas due to human disturbance [16, 17] or prey declines [e.g., 18, 19] and deteriorating conditions on the breeding grounds [20, 21]. Understanding the specific challenges that populations confront and where they occur is fundamental to effective management.

Whimbrels (*Numenius phaeopus*) are large holarctic waders that migrate over large geographic areas [22], are capable of extreme flights [23, 24] and utilize relatively few staging areas during migration [25, 26]. The two populations (Mackenzie Delta and Hudson Bay) that utilize the Western Atlantic Flyway winter together along the northern Coast of South America but use separate autumn migration routes that differ in exposure to tropical cyclones [27]. Whimbrels exhibit delayed recruitment and have low reproductive potential [12]. A recent assessment suggests that adult mortality rates exceed sustainable levels [28]. Surveys within a spring staging area [29] and within the primary winter grounds [30] indicate that at least one of the populations using the Western Atlantic Flyway has experienced significant declines over the past three decades.

Identifying where and when populations spend their time is a first step toward understanding exposure to hazards and developing conservation strategies to overcome them. In this study, we used satellite transmitters to track adult Whimbrels from two breeding populations to explore the structure of their annual cycles. Our objectives included (1) documenting population-specific seasonal schedules, (2) mapping autumn and spring migration pathways for the two populations, (3) delineating significant migratory staging area and stopover sites and (4) identifying locations of overlap between the populations.

## Methods

### Field methods

We captured 34 whimbrels (Table 1) between 2008 and 2018 on migratory staging areas along the lower Delmarva Peninsula in Virginia, USA (*n* = 13) (37.398° N, 75.865° W), along the coast of Georgia, USA (*n* = 8) (31.148° N, 81.379° W), along the Acadian Peninsula in New Brunswick, Canada (*n* = 3) (47.973° N, 64.509° W) as well as on the nesting ground near the Mackenzie River, Northwest Territories, Canada (*n* = 10) (69.372° N, 134.894° W). All birds were aged as adults by plumage [31, 32] and were banded with United States Geological Survey tarsal bands and coded leg flags. Sex of captured birds was not determined.

**Table 1:** Summary information for adult Whimbrels tracked with satellite transmitters throughout the Western Atlantic Flyway (2008–2018).

Whimbrel	Transmitter size (g)	Breeding location	Spring	Breeding	Autumn	Winter
040166	9.5	Hudson Bay	0	0	1	0
050121	9.5	Hudson Bay	1	1	1	1
050122	9.5	Hudson Bay	0	0	1	0
074854.2	9.5	Hudson Bay	2	2	3	3
084206	9.5	Hudson Bay	2	2	2	2
085947	9.5	Hudson Bay	1	1	0	0
088039	9.5	Hudson Bay	1	1	1	0
088040	9.5	Hudson Bay	0	0	1	1
088041.1	9.5	Hudson Bay	1	1	0	0
088042.2	9.5	Hudson Bay	1	1	1	0
088044	9.5	Hudson Bay	1	1	1	0
088045	9.5	Hudson Bay	1	1	1	1
088046	9.5	Hudson Bay	0	0	1	0
098354	9.5	Hudson Bay	1	1	2	1
105874	9.5	Hudson Bay	4	4	4	4
117299	9.5	Hudson Bay	2	2	2	1
117300	9.5	Hudson Bay	2	1	1	1
123494	9.5	Hudson Bay	1	1	1	0
128483	5	Hudson Bay	1	1	1	0
074854.1	9.5	Mackenzie River	2	2	2	2
074854.3	9.5	Mackenzie River	1	2	2	1
088043.1	9.5	Mackenzie River	1	0	1	1
088043.2	9.5	Mackenzie River	4	4	4	4
103520	5	Mackenzie River	2	2	2	2
103521	5	Mackenzie River	1	1	1	1
103522.1	9.5	Mackenzie River	2	2	2	2
103522.2	5	Mackenzie River	1	2	2	2
105875	9.5	Mackenzie River	1	0	0	0
123745	5	Mackenzie River	1	2	2	1
123746	5	Mackenzie River	1	1	1	1
123748	5	Mackenzie River	2	2	2	2
133734	5	Mackenzie River	0	1	1	1
133735	5	Mackenzie River	4	5	5	4
133736	5	Mackenzie River	0	1	1	1

Numbers denote how many years for which each individual was tracked during a given season.

We fitted all birds with satellite transmitters called Platform Transmitter Terminals (PTTs) using a modification of the leg-loop harness [33, 34]. Instead of elastic cord, we used Teflon^®^ ribbon (Bally Ribbon Mills, Bally, Pennsylvania, USA) that was fastened with brass rivets or crimps [23]. We glued transmitters to a larger square of neoprene to elevate it above the body and prevent the bird from preening feathers over the solar panels. The transmitter package was below 3% of body mass (measured at the time of deployment ($\bar{x}$ = 484.5±17.1) for all individuals tracked in this study. The PTTs used in this study were 9.5 g PTT-100 (*n* = 24) or 5.0 g PTT-100 (*n* = 10) solar-powered units produced by Microwave Telemetry, Inc. (Columbia, Maryland, USA).

### Tracking

Birds were located using satellites of the National Oceanic and Atmospheric Administration and the European Organization for the Exploitation of Meteorological Satellites with onboard tracking equipment operated by Collecte Localisation Satellites (CLS America, Inc., Largo, Maryland, USA) [35]. Transmitters were programmed to operate with a duty cycle of 24 h off and 5 h on (*n* = 10) or 48 h off and 10 h on (*n* = 24) and collected 1–34 ($\bar{x}$ = 5.48±0.07) locations per cycle. Locations in latitude and longitude decimal degrees, date, time, and location error were received from CLS America within 24 h of satellite contact with PTTs. Locations were estimated by the Advanced Research and Global Observation Satellite (ARGOS) system (www.Argos-system.org), which uses a Doppler shift in signal frequency and calculates a probability distribution within which the estimate lies. The standard deviation of this distribution gives an estimate of the location accuracy and assigns it to a “location class” (LC): LC3 = <150 m, LC2 = 150–350 m, LC1 = 350–1000 m, LC0>1000 m, LCA = location based on 3 messages and has no accuracy estimate, LCB = location based on 2 messages and has no accuracy estimate, and LCZ = location process failed. We used LC classes 1–3 to determine whimbrel locations.

### Seasonality

We used tracking data to subdivide the annual cycle into four seasons, including autumn migration, winter, spring migration, and breeding. Although migration may include fueling on breeding or winter territories before departure [36], here we consider the onset of migration to be when birds made decisive movements away from winter or breeding territories. In order to identify these breakout movements, we used locations on the breeding and winter territories to develop centroids and consider the first arrival or departure movement to be the first location that exceeded 2 standard deviation units beyond the mean of movements around centroids. We consider dates of arrival and departure to coincide with these locations during times when the transmitter was active. To account for cases where departure and arrival times occurred outside the transmitter’s duty cycle, we calculated the speed between the arrival or departure location and the last or next flight location. If the speed was less than 2 SD below the mean whimbrel flight speed ($\bar{x}$ = 14.7±0.3 m/s, n = 45), we interpolated arrival and departure times using the mean whimbrel flight speed and great circle distance between the two points. Departure was abrupt and we recorded no “false starts” of birds leaving breeding and winter areas and then returning before resuming migration. We delineated the length of each season, in days, for individuals based on their dates of arrival and departure to and from breeding and wintering grounds each year, and calculated summary statistics across all individuals and years.

### Migration pathways

We used tracking data to delineate migratory pathways during autumn and spring for each individual. We considered pathways to include the route traveled between the departure location from a stationary period and the arrival location to the next stationary period, but did not include local movements to and from roosting and foraging locations at stopover and staging sites. Due to the duty cycle of the transmitters our dataset had temporal gaps in coverage. We filled these gaps using continuous-time correlated random walk (CRAWL) models [37, 38] that allowed us to interpolate a pathway for each individual. We measured autumn and spring migration lengths (km) along the interpolated pathway from the departure to the arrival location. We estimated the duration of the migration as the elapsed time between departure and arrival including any stops along the route. We estimate the migration speed toward migratory destinations as the migration length/elapsed time (km/d). Centroids and distances between departure and arrival locations were calculated with the geosphere package [39] in Program R [40].

### Staging and stopover locations

We used tracking data to delineate staging and stopover areas used by whimbrels during migration. We adopted the terminology of Warnock [41] to differentiate staging areas and stopover areas. We consider staging areas to be annually used destinations that 1) have high resource densities to allow for rapid refueling rates, 2) have long distances (>1,000 km) to the next destination, 3) support large portions of the population where 4) birds stay for long (weeks) periods and 5) exhibit high site fidelity between years. We consider stopover areas to be unplanned “emergency” areas where conditions force birds to stop that 1) support small portions of the population where 2) birds stay for short (days) periods or 3) exhibit low site fidelity between years. We associated stopover areas with their upstream staging area. We recorded the number of stopover areas used by each individual during all years and associated with each staging area. We used the same approach as above for breeding and winter areas to determine the length of stay within both staging area and stopover sites.

### Statistics

We developed descriptive statistics including means and standard errors for seasons (initiation dates and duration), migration (distance, duration and migration speed), staging areas (arrival dates and duration of stay) and stopover areas (number per migration leg and duration of stay) and compared these metrics between populations and seasons as appropriate using two-tailed t-tests with Bonferroni-corrected p-values. Several birds made more than one migration in different years and we consider these to be independent samples.

### Ethics statement

All birds were captured and handled under the Institutional Animal Care and Use Committee protocol IACUC-2017-04-18-12065 of The College of William and Mary, Environment Canada Animal Care Committee protocols EC-PN-12-006, EC-PN-13-006, EC-PN-14-006, Mount Allison University Animal Care Committee protocol 15–14, and the Government of the Northwest Territories Wildlife Care Committee protocol NWTWCC2014-007.

## Results

### Phenology

The temporal structure of the annual cycle varied between populations, with the Mackenzie Delta population exhibiting earlier dates of seasonal transition (Table 2). The Mackenzie Delta population arrived on the breeding grounds an average of 4 days earlier, left for autumn migration 15 days earlier, arrived on the winter grounds 19 days earlier and left the winter grounds 14 days earlier compared to the Hudson Bay population. Although the structure of the annual cycle was generally consistent between populations, some key differences are apparent. Hudson Bay birds were resident on the breeding grounds for a longer period (46.4 ± 3.3 compared to 36.2 ± 4.0 days) and had a shorter period (47.1 ± 3.5 compared to 53.7 ± 2.1 days) devoted to spring migration when compared to Mackenzie Delta birds. Although the longer period of residency on the breeding grounds may reflect birds that staged in place prior to autumn migration this possibility does not fully account for the differences in schedules. The span between arrival on the breeding grounds and arrival on the winter grounds was 12 days longer for Hudson Bay birds (107 vs 91 days) compared to Mackenzie Delta birds suggesting some fundamental differences in the summer and autumn seasons. Winter dominated the annual cycle for both populations averaging around 7.3 months or 60% of the year for both the Mackenzie Delta and Hudson Bay populations.

**Table 2:** Mean initiation dates of seasons for whimbrels from Mackenzie Delta and Hudson Bay breeding populations as determined by satellite tracking between 2008 and 2018.

	Mackenzie Delta		Hudson Bay			
Season	*n* (x)	Mean ± SE	*n* (x)	Mean ± SE	*t*	*p*
Breeding	10 (18)	31 May ± 2.5	15 (21)	3 June ± 4.24	0.565	0.576
Autumn migration	13 (26)	7 July ± 2.0	11 (17)	22 July ± 3.0	3.940	<0.001
Winter	13 (25)	30 August ± 3.3	9 (15)	18 September ± 4.1	3.446	0.002
Spring migration	13 (21)	6 April ± 1.6	7 (10)	20 April ± 2.8	4.427	<0.001

Sample sizes represent number of individuals (total number of seasons) on which initiation dates were based.

### Migratory pathways

**Fig 1:**
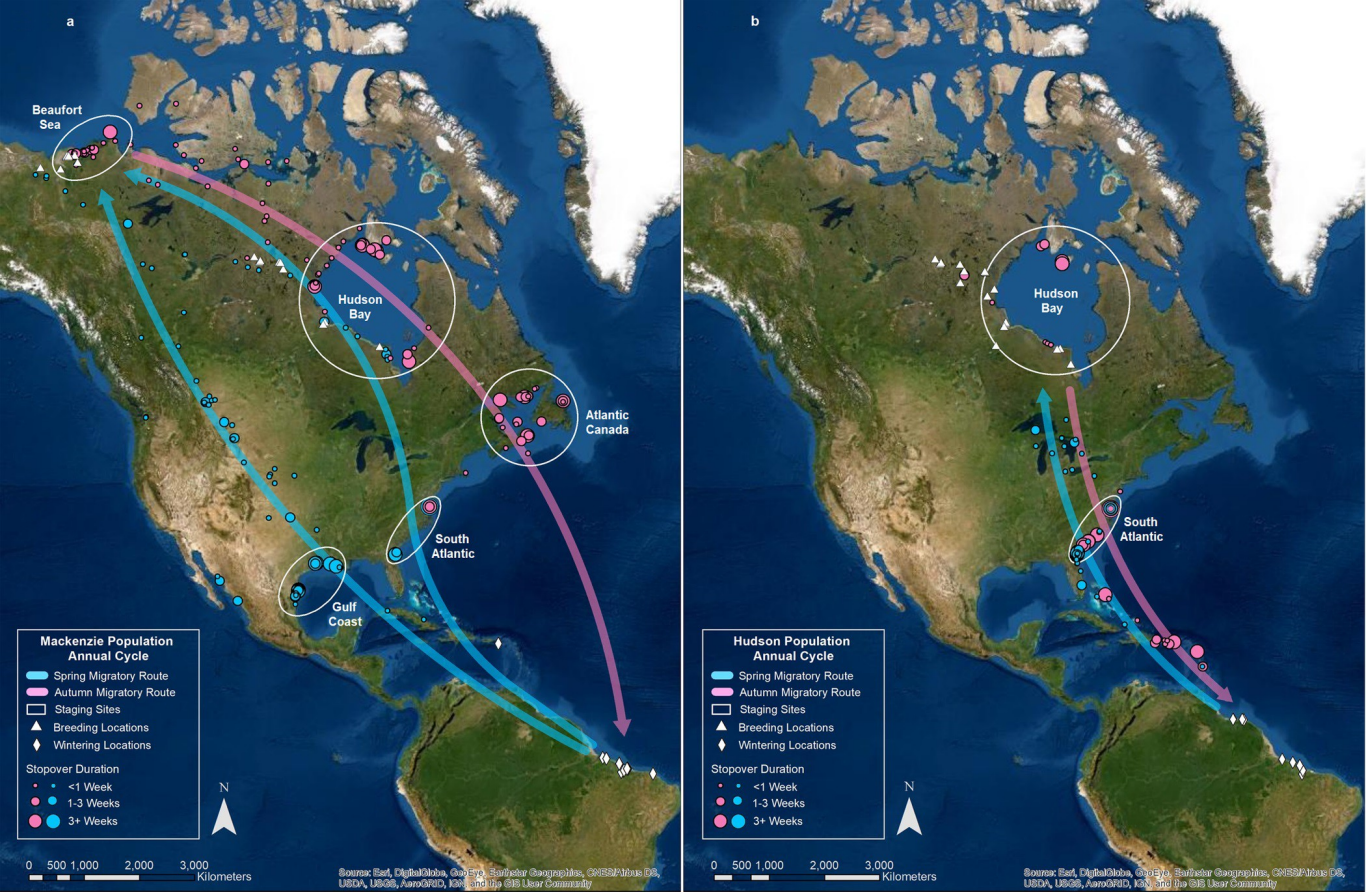
Stylized, Composite Maps of The Annual Cycle for the Mackenzie Delta (1a) and the Hudson Bay (1b) Whimbrel Populations. Information represents a synthesis of multiple years of tracking data. Dots and diamonds represent centroids of locations from periods when birds were not flying. Figure was created by one of the authors using ArcGIS 10.4.1. Basemap provided by: ESRI, Digital Globe, GeoEye, Earthstar Geographics, CNES/Airbus DS, USDA, USGS, AeroGRID, IGN, and the GIS User Community.

Adult birds from the Mackenzie Delta and Hudson Bay breeding populations follow distinctly different migratory pathways such that they are separated during much of the year. Following the breeding season, birds from Mackenzie Delta staged around the Beaufort Sea, flew east across the continent reaching terminal staging areas primarily in Atlantic Canada but also in Hudson and James Bays (Fig 1A). Nearly all birds made transoceanic flights to winter territories along the east coast of northern South America. An exception to this pattern is a bird that staged in Hudson Bay and made direct flights to a winter territory on St. Croix or to the South Atlantic staging area before flying to St. Croix. From the winter grounds, birds used two major routes to reach the breeding grounds including a northern route with staging on the south Atlantic Coast and a southern route with staging on the western Gulf Coast. Birds staging along the Atlantic Coast flew toward the Great Lakes and then west to the Mackenzie Delta. Birds staging along the Gulf Coast flew directly to the Mackenzie Delta over the Great Plains and skirting the eastern flank of the Rocky Mountains. An exception to this southern route is a bird that flew across northern Mexico to stage on the Gulf of California for two consecutive years and flew north along the Pacific Flyway to the Mackenzie Delta.

In contrast to the Mackenzie Delta population, whimbrels breeding within the Hudson Bay lowlands flew similar routes to and from the breeding and winter grounds (Fig 1B). Following the breeding season, birds either staged within the breeding grounds or moved to Hudson and James Bays to stage before flying south to stage along the Atlantic Coast. Birds flew from the Atlantic Coast across the Caribbean Basin to the northern coast of South America. Many birds made landfall on the coast of South America well west of their winter grounds and slowly moved east until reaching their final destination. From the winter grounds, birds retraced their autumn route north to stage along the Atlantic Coast before flying over the Great Lakes and on to Hudson Bay.

Differences in the distance between breeding and winter grounds and migratory routes resulted in differences between populations in overall migration distance, duration and the migration speed toward spring or autumn destinations (Table 3). Within populations, spring and autumn migration distances were not distinguishable for Hudson Bay (t_16_ = 0.9, p = 0.38) or Mackenzie Delta (t_35_ = -0.3, p = 0.75) despite different seasonal pathways for the latter. However, both spring (t_23_ = 4.1, p < 0.001) and autumn (t_28_ = 4.4, p < 0.001) distances were different between populations. The duration of migration was similar between populations during the autumn (t_28_ = -0.5, p = 0.64) but differences were notable during spring (t_23_ = 1.9, p = 0.06). Though mean migration speed was higher for both populations during spring (Table 3), the difference was not significant (Mackenzie Delta: t_35_ = -1.8, p = 0.08 and Hudson Bay: t_16_ = -2.0, p = 0.06). Mackenzie Delta migration speed was higher than Hudson Bay during autumn (t_28_ = 2.3, p = 0.03) but not spring (t_23_ = 1.1, p = 0.28). For all whimbrels (both populations combined), migration speed was significantly (t_53_ = 2.4, p = 0.02) higher during the spring (206.9±10.47) compared to the autumn (173.8±8.79).

**Table 3:** Autumn and spring migration statistics for whimbrels from the Mackenzie Delta and Hudson Bay breeding populations as determined by satellite tracking (2008–2018).

Migration	*n* (x)	Distance (km)	Duration (d)	Migration Speed (km/d)
Mackenzie Delta				
Autumn	12(21)	11,332±333.8	64±3.6	186±10.5
Spring	9(16)	11,518±487.7	55±2.1	216±12.1
Hudson Bay				
Autumn	5(9)	8,989±236.3	68±9.0	145±11.7
Spring	6(9)	8,581±389.2	47±3.5	191±19.5

Sample sizes represent number of individuals (total number of seasons) on which values were based.

### Staging areas and stopover sites

We identified five migratory staging areas used by whimbrels (Table 4). All areas were used during at least one season by the Mackenzie Delta population but only two were used by the Hudson Bay population. Fidelity to staging areas was very high with 98% (N = 52) of birds that used a specific areas in one year also using the area during the following year. The single exception was a Mackenzie Delta bird that staged around Hudson Bay one year but over flew the area in the following year. The structure of use was simple and straightforward. During the autumn Mackenzie Delta birds staged around the Beaufort Sea and either flew directly (31.6% of events, n = 19) to Atlantic Canada and then on to South America or to Hudson Bay (68.4% of events). Birds that staged in Hudson Bay either flew straight to South America (30.1% of events, n = 13), on to Atlantic Canada and then on to South America (46.2% of events) or to the South Atlantic and on to South America (23.1% of events). Hudson Bay birds staged around the Bay and either made attempts to fly nonstop (15.4% of events) to South America or flew (84.6% of events) to the South Atlantic Coast of the United States to stage before flying to South America. During the spring both populations used single staging areas between winter and breeding areas with Mackenzie Delta birds being split between staging areas along the South Atlantic Coast and the Gulf Coast of the United States and Hudson Bay birds using the South Atlantic Coast. Within staging areas, birds were generally scattered. Exceptions were the South Atlantic Coast where birds were concentrated along the Delmarva Peninsula in Virginia and along the coasts of South Carolina and Georgia and along the Gulf Coast where birds were concentrated along the northern coast or Texas and western Louisiana and along the northern coast of Mexico. Arrival times on staging areas and duration of events varied between populations and areas (Table 4). Although notable, most differences were not significant likely due to low statistical power. An exception is that Mackenzie Delta birds arrive in spring along the South Atlantic Coast significantly (t_19_ = 4.4, p < 0.001) earlier than Hudson Bay birds.

**Table 4:** Use of staging and stopover sites by whimbrels from the Mackenzie Delta and Hudson Bay breeding populations as determined by satellite tracking (2008–2018).

Staging Areas	*n* (x)	Arrival Date	Staging Duration (d)	Post-staging Stopovers (N)	Stopover Duration (d)
Mackenzie Delta					
Autumn					
Beaufort Sea	10 (17)	04 Jul±1.65	7.6±1.25	1.3±0.31	1.8±0.40
Hudson Bay	10 (16)	15 Jul±1.82	18.0±2.58	0.1±0.08	0.6±0.00
Atlantic Canada	12 (19)	29 Jul±2.41	19.3±2.52	0	0
Spring					
South Atlantic	3(5)	17 Apr±5.84	35.0±6.53	2.8±1.18	1.0±0.37
Gulf Coast	9(16)^1^	11 Apr±2.19	25.0±2.51	2.4±0.75	2.5±0.45
Hudson Bay					
Autumn					
Hudson Bay	5 (7)	18 Jul±9.25	15.5±3.66	1.8±0.75	9.5±3.94
South Atlantic	8 (13)^1^	02 Aug±4.11	28.4±1.79	1.0±0.02	12.4±2.94
Spring					
South Atlantic	7(10)	29 Apr±3.77	29.2±3.96	1.2±0.18	1.9±0.91

Sample sizes represent number of individuals (total number of seasons) on which values were based. Values represent means±SE. Stopovers reflect events recorded during flights moving away from staging (see Fig 1A and 1B).

^1^Tracking of one bird ended within staging area so sample size for duration is one less.

We documented locations where birds stopped for short periods of time along all migration routes except the transoceanic route from Atlantic Canada to South America, where potential stopover sites do not exist (Fig 1A and 1B). The mean number of stopovers per individual and the stopover length varied between seasons and migration legs (Table 4). Notable clusters of stopover sites include the upper Great Plains for Mackenzie Delta birds during the spring, the Great Lakes for Hudson Bay birds during the spring, around the Beaufort Sea and associated waterways for Mackenzie Delta birds during autumn and throughout the Caribbean Basin in the autumn where Hudson Bay birds are put down by storms [27].

## Discussion

Tracking whimbrels with satellite transmitters has revealed differences in the annual cycle between the Mackenzie Delta and Hudson Bay breeding populations in terms of both the timing of transitions between seasons and space use. Mackenzie Delta whimbrels left the breeding grounds, arrived on winter grounds and left winter grounds earlier than whimbrels breeding around Hudson Bay. Mackenzie Delta whimbrels followed a long loop migration pathway with different autumn and spring routes while Hudson Bay whimbrels followed a shorter more direct pathway between breeding and winter grounds with similar autumn and spring routes. Both populations had higher rates of migration during spring compared to autumn, though the difference was not significant. More rapid migration was facilitated by a very low number of short stopovers between terminal staging areas and the breeding grounds. The two populations share the winter grounds along the northern coast of South America. The only other area of consistent overlap during the annual cycle appears to be along the South Atlantic Coast in the spring where a portion of the Mackenzie Delta birds mix in to stage with Hudson Bay birds.

Tracking information has clarified patterns of space use. Historically, we believed that North America supported two whimbrel breeding populations including an eastern population (Hudson Bay) that migrated along the Western Atlantic Flyway and a western population (Mackenzie Delta and Alaska) that migrated along the Pacific Flyway [42]. Through this lens we interpreted birds that appeared in Atlantic Canada in autumn as migrants from Hudson Bay and birds that appeared later along the South Atlantic Coast as the same birds. Hudson Bay birds were believed to leave the breeding grounds, fly to Atlantic Canada to feed on berries in late summer, fly to the South Atlantic Coast to feed on fiddler crabs, continue down the coast to Florida where they would depart and fly over the Caribbean Basin to South America [43]. We now know that North America supports at least three whimbrel breeding populations including Hudson Bay, Mackenzie Delta and Alaska. Hudson Bay birds fly south from the breeding grounds to stage along the South Atlantic Coast or directly to South America [25]. The birds that stage in Atlantic Canada are from the Mackenzie Delta. These birds stage for an extended period and make a nonstop, transoceanic flight to South America.

Birds using different migration routes and staging areas face different sets of constraints that shape respective schedules [1]. Mackenzie Delta whimbrels left the breeding grounds more than two weeks earlier than Hudson Bay whimbrels. Whether the early departure reflects the need to take advantage of a window of favorable tail winds to reduce energy expenditure on their flight across the continent, the need to match their arrival in Atlantic Canada to the ripening of the rich berry community required to fuel their transoceanic flight [22, 44], the need to take advantage of a window of favorable winds in their flight across the Atlantic Ocean [45, 46] or the need to establish winter territories before the arrival of competitors from Hudson Bay [47, 48] remains unclear. Hudson Bay whimbrels left breeding areas later and spent longer periods within terminal staging areas despite flying shorter distances to South America. It is possible that contending with constraints imposed by tides [49] and digestive bottlenecks [50] associated with consuming fiddler crabs require longer periods to achieve leaving condition compared to birds in Atlantic Canada feeding in non-tidal habitats on easily digestible fruits. It is also possible that birds from Hudson Bay delayed their flight across the Caribbean Basin to avoid the peak of hurricane activity [27].

The short Arctic season places high pressure on migrants to arrive before or soon after habitats thaw for breeding [2, 51]. Birds that arrive early on the breeding grounds may have more time to replenish nutrient reserves [52] attain the best territories [53], have a longer window to renest in the event of early failure [54] and tend to have higher young survival [55, 56]. Throughout their annual schedule, Mackenzie Delta and Hudson Bay whimbrels reached their greatest synchrony (mean arrival differ by 4 days) in their arrival on the breeding grounds. Since Mackenzie Delta birds must complete a longer flight (mean distance traveled differs by 2937 km) between the South Atlantic Coast and their breeding grounds compared to Hudson Bay they may require a larger fuel load at departure. Mackenzie Delta birds appear to insure they reach an adequate leaving condition by a “date certain” by leaving the winter grounds earlier, arriving along the South Atlantic Coast earlier and staging longer than Hudson Bay birds. Of all the staging periods throughout the annual cycle, the terminal spring staging event is the longest for both populations. Presumably, this extended period allows for preparations to both complete their terminal flight to the breeding grounds and to arrive in adequate breeding condition.

A wide variety of shorebirds have been shown to achieve a higher migration speed toward the breeding grounds in the spring compared to away from the breeding grounds in autumn [57] suggesting high selective pressure for birds to be present for the onset of the short breeding season compared to the long winter season. Both Mackenzie Delta and Hudson Bay whimbrels migrated faster during spring compared to autumn with differences between the two seasons averaging 30 and 46 km/d faster for the two populations respectively. Both populations were able to achieve higher migration speeds during the spring by having fewer and shorter stopovers *en route*. This pattern is consistent with predictions of optimal migration [58]. Increasing fueling rates and/or reducing stopover events is often more beneficial since increasing flight speed requires greater power and fuel consumption, thus compromising condition upon arrival.

Although whimbrels range over large geographic areas during the course of their annual cycles they are capable of long flights [23, 24] and appear to use this capability to reach and exploit a small set of staging areas for which they have high fidelity. The consistent use of relatively few areas suggests that these areas are integral to their annual cycle and have high conservation value. Of particular significance appears to be terminal spring staging areas including the South Atlantic and Gulf Coasts where birds stage for long periods before moving on to breeding grounds. These two areas appear to be strategically important population bottlenecks that should be prioritized for conservation.
